# Application of artificial neural network for predicting the performance of CO_2_ enhanced oil recovery and storage in residual oil zones

**DOI:** 10.1038/s41598-020-73931-2

**Published:** 2020-10-23

**Authors:** Hung Vo Thanh, Yuichi Sugai, Kyuro Sasaki

**Affiliations:** Department of Earth Resources Engineering, Faculty of Engineering, Kyushu University, 744 Motooka, Nishi-ku, Fukuoka, 819-0395 Japan

**Keywords:** Climate-change adaptation, Climate-change mitigation, Energy and society, Sustainability, Carbon capture and storage, Fossil fuels

## Abstract

Residual Oil Zones (ROZs) become potential formations for Carbon Capture, Utilization, and Storage (CCUS). Although the growing attention in ROZs, there is a lack of studies to propose the fast tool for evaluating the performance of a CO_2_ injection process. In this paper, we introduce the application of artificial neural network (ANN) for predicting the oil recovery and CO_2_ storage capacity in ROZs. The uncertainties parameters, including the geological factors and well operations, were used for generating the training database. Then, a total of 351 numerical samples were simulated and created the Cumulative oil production, Cumulative CO_2_ storage, and Cumulative CO_2_ retained. The results indicated that the developed ANN model had an excellent prediction performance with a high correlation coefficient (R^2^) was over 0.98 on comparing with objective values, and the total root mean square error of less than 2%. Also, the accuracy and stability of ANN models were validated for five real ROZs in the Permian Basin. The predictive results were an excellent agreement between ANN predictions and field report data. These results indicated that the ANN model could predict the CO_2_ storage and oil recovery with high accuracy, and it can be applied as a robust tool to determine the feasibility in the early stage of CCUS in ROZs. Finally, the prospective application of the developed ANN model was assessed by optimization CO_2_-EOR and storage projects. The developed ANN models reduced the computational time for the optimization process in ROZs.

## Introduction

Carbon capture, utilization, and storage (CCUS) is the potential solution to slow down greenhouse gas emission and climate change^1,2^. CO_2_ could be stored in many possible formations such as saline aquifers, depleted hydrocarbon reservoirs, depleted fractured shale formations, fractured basement reservoirs, and deep ocean formations^3–6^. Currently, Residual Oil Zones (ROZs) have been considered as promising formations for long-term geological CO_2_ storage^7^. ROZs are the reservoirs in which the oil is at or migrate closer to the residual oil saturation^8^. ROZs are the most optimum reservoirs to store CO_2_^9,10^.

Moreover, many similar studies have been investigated the feasibility and promising of CO_2_ EOR and storage in ROZs. There are many different types of ROZs in the term of origin and evolution^10^. Harouaka et al.^11^ divided the ROZs into types; brownfield ROZs are the main pay zone (MPZ) below the oil–water contact of reservoirs and the greenfield is the only residual oil zone or not associated with normal oil reservoirs. Unfortunately, ROZs are not official for oil exploitation because conventional techniques cannot produce oil at these formations^7^.

However, there are several studies that demonstrated the success of oil production in ROZs using CO_2_-EOR method^8,11–13^. In particular, the Permian basin with the active ROZs employed the CO_2_ injection in a number of field projects, but the actual evaluation of ultimate oil recovery is not yet investigated^14^. Also, the CO_2_ storage capacity in ROZs capacity is still preliminary estimates with large uncertainties^15^. This is because of the limitation of characterization data and existing geological uncertainties in ROZs. CO_2_-EOR and storage in MPZ have been presented in various studies^16–21^. Ettehadtavakkol et al.^16^ proposed the framework for optimal design to rank CO_2_-EOR and storage candidates. Ahmadi et al.^17^ used the numerical simulation to perform CO_2_ sequestration and EOR into the pay zone and aquifer. Zhang et al.^18^ calculated the CO_2_ storage capacity in the H-59 block of Jilin oilfield China. They investigate the trapping mechanism and CO_2_ plume shape using the well pattern, reservoir heterogeneity and injected CO_2_ amount. Ampomah et al.^19^ proposed the integrated workflow based on the uncertainty quantification method and the artificial neural network optimization approach to co-optimize the CO_2_ storage and EOR in the Farnsworth Unit oil field in Texas. Dai et al.^20^ employed Monte Carlo (MC) simulations for the quantification uncertainty of CO_2_ sequestration potential within an active EOR project in the Morrow reservoir at the Farnsworth Unit, Texas. Hill et al.^21^ stated that geologic CO_2_ storage coupling EOR provides the benefits to improve oil recovery, which offsets major capital costs of capture and storage facility.

Regarding the CO_2_-EOR in ROZs, many type of researches were conducted to prove the potential oil recovery^22–25^. Koperna et al.^22^ demonstrated that the CO_2_ flooding is potential for the oil field development plan in ROZs of the Permian Basin. Honarpour et al.^23^ utilized the laboratory data and compositional CO_2_ flood simulation to evaluate the oil recovery of Residual oil zones in the Seminole San Andres Unit. They tried to understand the rock fluid characterization and model the complexity in ROZs. Bergmo et al.^24^ conducted the simulation of CO_2_-EOR on water-flooded oil reservoirs underlying the paleo residual oil zone to estimate the potential oil recovery on ROZs. Stewart et al.^25^ carried out the simulation study to investigate the feasibility of CO_2_ injection in ROZs of Pierce Oil Field, Central North Sea. These authors confirmed that the CO_2_-EOR could produce the low carbon intensity crude oil in the mature field.

By reviewing the literature, studies on coupled CO_2_-EOR and storage in ROZs are minimal. Ren and Ducan ^26^ used the numerical simulator (Eclipse-300) to evaluate the performance of CO_2_-EOR and storage by adjusting injection strategies, well configurations, and injection pattern in the real San Andreas ROZs reservoir. This work contributed to better understand for the future development of CO_2_ storage and EOR in ROZs.

Jamali and Ettehadtavakkol et al.^27^ conducted the field scale modelling for MPZ and ROZs San Andres Unit in the Permian Basin to assess the potential Carbon storage and ability to reduce leakage in ROZ. Recently, Chen and Pawar^28^ developed the novel method using numerical simulation and statistical analysis in residual oil zones of Goldsmith-Landreth San Andres. These authors demonstrated the effectiveness of the predictive empirical model using machine learning could provide for capacity assessment and optimization of CO_2_-EOR and storage. However, the composition reservoir simulation is taking a long time processing for engineering problems such as sensitivity analysis and optimization process.

Also, the simulation work needs a large number of data such as seismic, well log, and core data. These issues could be solved by an Artificial Neural Network (ANN) to create the smart proxy model for the predictive purpose. ANN can be employed as an alternative solution for complicated problems in the reservoir engineering^29^. There are many studies to use ANN in petroleum engineering, such as the screening enhance oil recovery “method”^30^, assisted history matching^31^, estimation dew point pressure^32^, drilling engineering^33^ ,etc. In the case of CO_2_ sequestration, Kim et al.^34^ used ANN for prediction storage efficiency in a saline aquifer. These authors stated that the ANN model is a robust tool for predicting the feasibility of CO_2_ sequestration with high accuracy. Moreover, Ahmadi et al.^35^ applied ANN for the prediction of the CO_2_ properties in carbon capture and sequestration operations. Besides, ANN models were used for the evaluation performance of the WAG process in CO_2_-EOR and sequestration projects^36,37^.

Furthermore, several studies were employed ANN-based proxies models for EOR projects^38,39^. These authors stated that the ANN expert system could propose the fast technical and economic assessment for EOR projects. Recently, You et al.^40^ proposed a robust framework that integrating the ANN and multi-objective optimizers to find the optimal solution for CO_2_-EOR and storage in the FWU field.

Regarding the application of machine learning tools to sever as fast proxy models of high-fidelity reservoir simulation using regression approach^41^, artificial neural network^42^. Besides machine learning approach was supported for other reservoir engineering problems such as history matching^43^, reservoir characterization^44^. These studies were demonstrated that the utilization of a machine learning approach would improve computational efficiency with complex issues in subsurface engineering.

However, ANN techniques have not been implemented to create predictive models to estimate the oil recovery performance and CO_2_ storage capacity for depleted reservoirs or ROZs. Thus, this study aims to propose the ANN models for generation predictive tools to evaluate the feasibility of CO_2_-EOR and storage with simply and reducing time-consuming compositional reservoir simulation for ROZs. Also, this study was coupled Particle Swarm Optimization and ANN system to speed up the optimization process of the CO_2_-EOR project.

To best of our knowledge, this work is the first to be adapted ANN model for a generation the robust predictive tools in CO_2_-EOR and storage in ROZs.

In sum, the main objectives of our work are the following:

- To create the predictive models for CO_2_-EOR and storage in ROZs.
- To generate the rapid tool reducing time-consumable compositional reservoir simulation.
- To validate the stability and accuracy of ANN models using the real ROZs field in the Permian Basin.
- To demonstrate the application perspective of ANN models for optimization CO_2_ injection process.

### Artificial neural networks model generation

**Figure 1:**
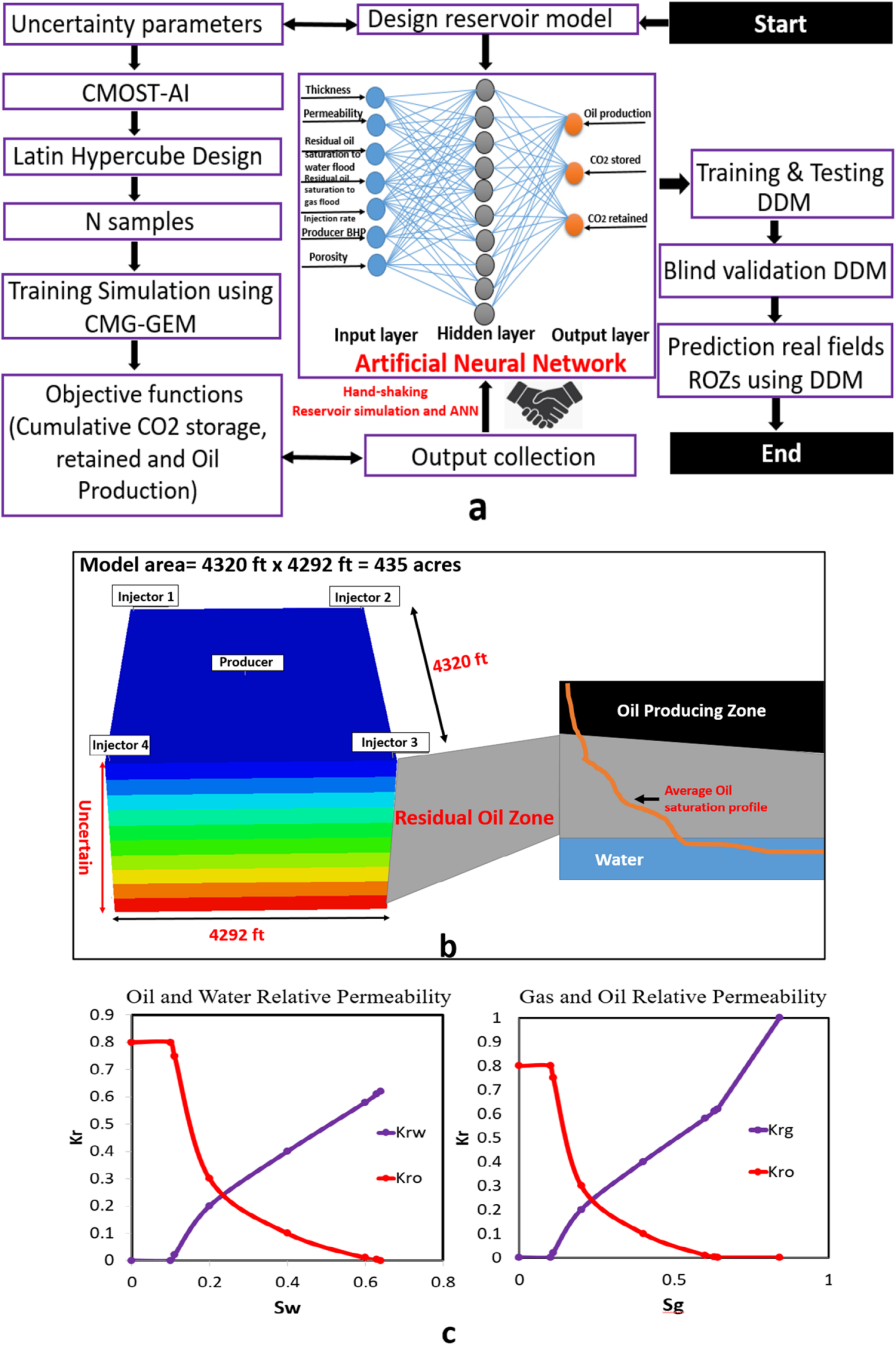
Data-driven modelling workflow, reservoir model, and rock-fluid properties. (**a**) The framework linked between the reservoir simulation and ANN for data-driven models. (**b**) The illustration of base case reservoir models and location of the Residual Oil Zone in the subsurface. (**c**) The behavior of relative permeability curves used for reservoir simulation.

Artificial Intelligence (AI)-neural networks are a common method for a generation of predictive models. AI-based reservoir simulation employed pattern recognition to teach reservoir performance to a computer^45^. Moreover, the data-driven model could be created a fast and accurate prediction instead of reservoir simulation. For this paper, the data-driven models are built to evaluate the CO_2_ storage capacity and oil field recovery in Residual Oil Zones. The workflow for the construction of the data-driven models is depicted in Fig. 1a. The procedure of this workflow is summarized as follows:

*Step 1*: *Design Simulation Model*. A 3D reservoir model was used CMG-GEM to simulate the CO_2_ injection process. The reservoir properties were referenced from Goldsmith-Landreth San Andres Unit (GLSAU) in the Permian Basin^46^. The five-spot well pattern scale was considered for this study. As depicted in Fig. 1a, the simulation model has 12,960 (36 × 36 × 10) grid cells. The area of the pattern is equal to 435 acres. The horizontal size is 4392 *ft* × 4390 *ft* in the I and J directions^7^. The reservoir thickness and other properties were considered as uncertainty parameters. The base case and uncertainty variables are summarized in Table 1.

**Table 1:** The uncertainty variables for generation the reservoir simulation samples.

Uncertainty parameters	Minimum	Maximum	Units
Porosity	0.05	0.25	–
Permeability	0.01	200	*mD*
Thickness	50	300	*ft*
Residual oil saturation to gas flood (S_org_)	0.1	0.2	–
Residual oil saturation to water flood (S_orw_)	0.2	0.4	–
Producer bottom hole pressure	100	1500	*psi*
CO_2_ injection rate	5	20	*MM scf/day*

In previous research, the reservoir thickness and rock properties were adopted from core data^47^. In this study, information for rock-fluid properties was adapted from GLSAU in the history matching model by Trentham et al.^48^. Figure 1c highlighted the relative permeability relationship for this work. The oil is supposed to compose of a total of 10 pseudo hydrocarbon components (C1, C2, C3, C4, C5, C6, C7–C13, C14–C20, C21–C28 and C29 +)^47^. The mole fractions for each component are following: 0.3577, 0.0584, 0.0597, 0.0536, 0.0358, 0.0116, 0.2282, 0.081, 0.0416 and 0.0724^47^.

**Figure 2:**
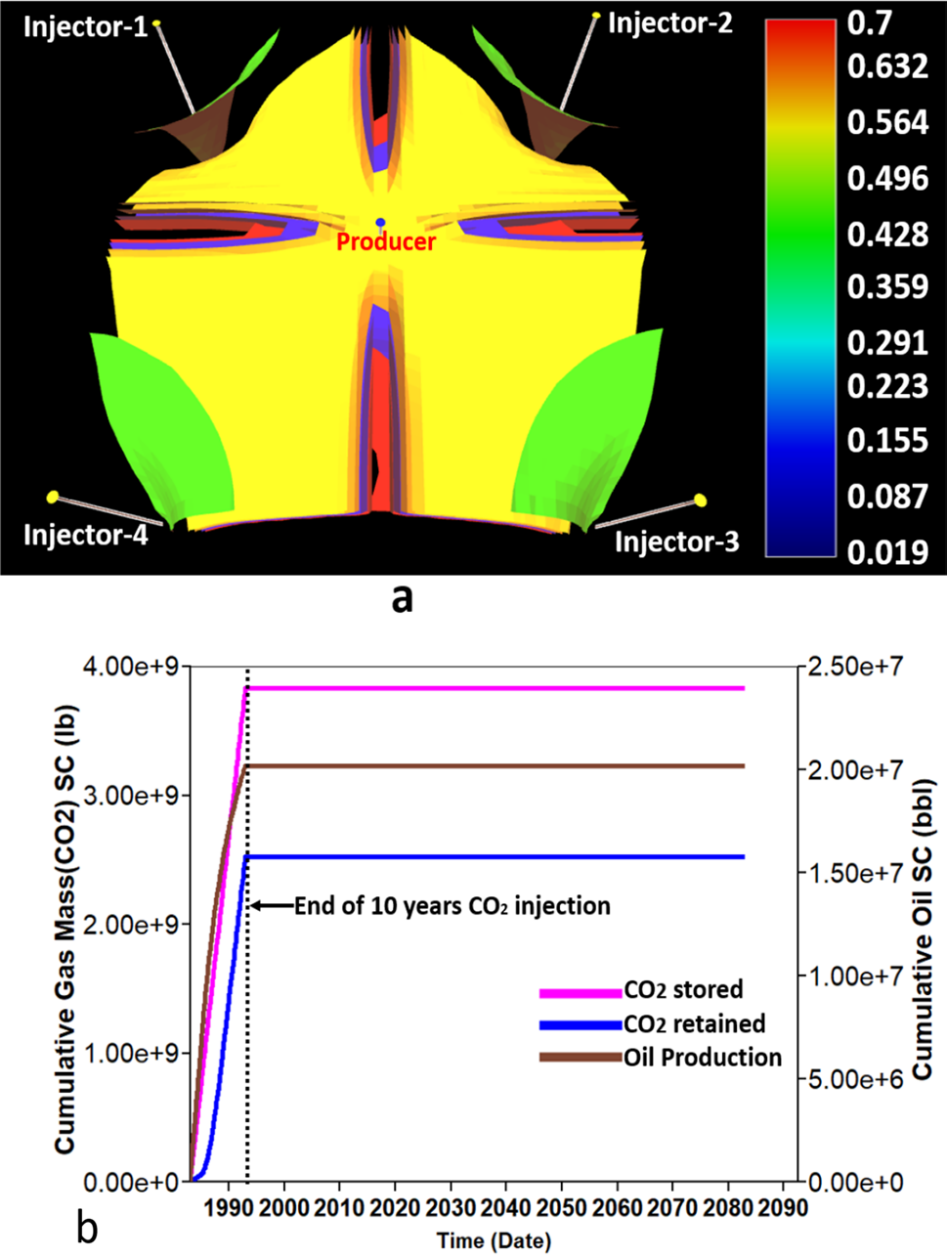
Base case reservoir simulation performance. (**a**) The iso-surface of oil saturation in the end of CO_2_ injection scheme. (**b**) The cumulative CO_2_ stored, CO_2_ retained, and oil production in the 10 years injection followed by 90 years post-injection period.

For this work, the continuous CO_2_ injection was conducted for 10 years injection phase followed by 90 years post-injection phase. Figure 2a highlights oil saturation at the end of 10 years of production. Figure 2b depicts the amount of CO_2_ stored and retained for the base case design model.

*Step 2: Define Uncertainty Parameters*. There is a lot of uncertainties factor in ROZs because these reservoirs are not common in the exploration and production process. Therefore, these uncertainties could be used for the data-driven model to evaluate the performance of oil production and CO_2_ storage capacity in ROZs. In this study, the uncertainty variables are listed in Table 1. The range of uncertainty parameters are followed the studies of Koperna and Kuuskraa^22^, Honarpour et al.^23^, Trentham et al.^49^, Harouaka et al.^11^, Aleidan et al.^50^, Trentham et al.^48^.

*Step 3*: *Latin Hypercube Design*. To create the training database, 351 simulation jobs were created by Latin Hypercube Design (LHD) using CMOST-AI which is an Artificial Intelligence package from CMG. This tool is a powerful package for sensitivity analysis, history matching, optimization, and uncertainty assessment. The main reason is considering LHD because it is the independence of the number of training samples from the uncertainty variables.

*Step 4*: *Conduct training simulation samples to collect inputs/outputs for the ANN models. *This step is an important process for the data-driven model. The compositional simulator CMG-GEM is used to conduct 351 simulation jobs. For each simulation job, the uncertainty variables (inputs) and the equivalent objective interests (outputs) were collected as the training database. The objective interests are cumulative Oil Production, the cumulative CO_2_ stored, and the cumulative CO_2_ retained in ROZs.

*Step 5*: *Create ANN model for objective functions. *In this study, ANN has utilized for a generation the data-driven model. Basically, the structure of a neural network consists of the input layer, output layer, and one or more hidden layers. Moreover, the neural network black-box of MATLAB was adapted to develop the predictive model. The training ANN process was performed using the Levenberg–Marquardt (LM) algorithm. This algorithm is supported by reducing the output error in all of the connection weights^34^. The input variables include the parameters listed in Table 1. The training data set is randomly divided into three main parts such as training, validating, and testing. During the ANN training process, the accuracy of the prediction output variables observed by investigating the cross plot of ANN predicted values and simulation results. R-squared values are considered for the evaluation ANN model. Also, the error of the training data and blind testing set are the second criteria to evaluate the ANN model. This model training will stop when three criteria are satisfied. First, the largest R-squared values are obtained, and second, the root mean square error (RMSE) is not decreased any more.

These constraints prevent the over-fitting issue and evaluating the performance of the data-driven model-based ANN^51^. The two decisive factors are calculated using formulas:

$$R^{2} = 1 - \frac{{\sum\nolimits_{i = 1}^{n} {(x_{i,sam} - x_{i,pred} )^{2} } }}{{\sum\nolimits_{i = 1}^{n} {(x_{i,sam} - \overline{x}_{i,sam} )^{2} } }}$$

$$RMSE = \sqrt {\frac{1}{n}\sum\nolimits_{i = 1}^{n} {(x_{i,sam} - x_{i,pred} )^{2} } }$$

where $x_{i,sam}$, $x_{i,pred}$, $\overline{x}_{i,sam}$ are data point from numerical simulation samples, the prediction values by neural network and the average of numerical sampling data, respectively.

*Step 6*: *Validation and field application of ANN model.* To employ the ANN model for prediction purposes. The 351 samples were used for training and blind testing purpose to ensure the stability of the data-driven model. Then, the data-driven model will be deployed in the five real ROZs fields from the Permian Basin. This step will ensure that the feasibility of data-driven in the real field application not only in CO_2_-EOR and storage but also in other science/engineering disciplines. The MATLAB equation of ANN model for prediction field data expressing as: *Result* = *net *(*matrix data*).

## Results

### Samples for ANN training

**Figure 3:**
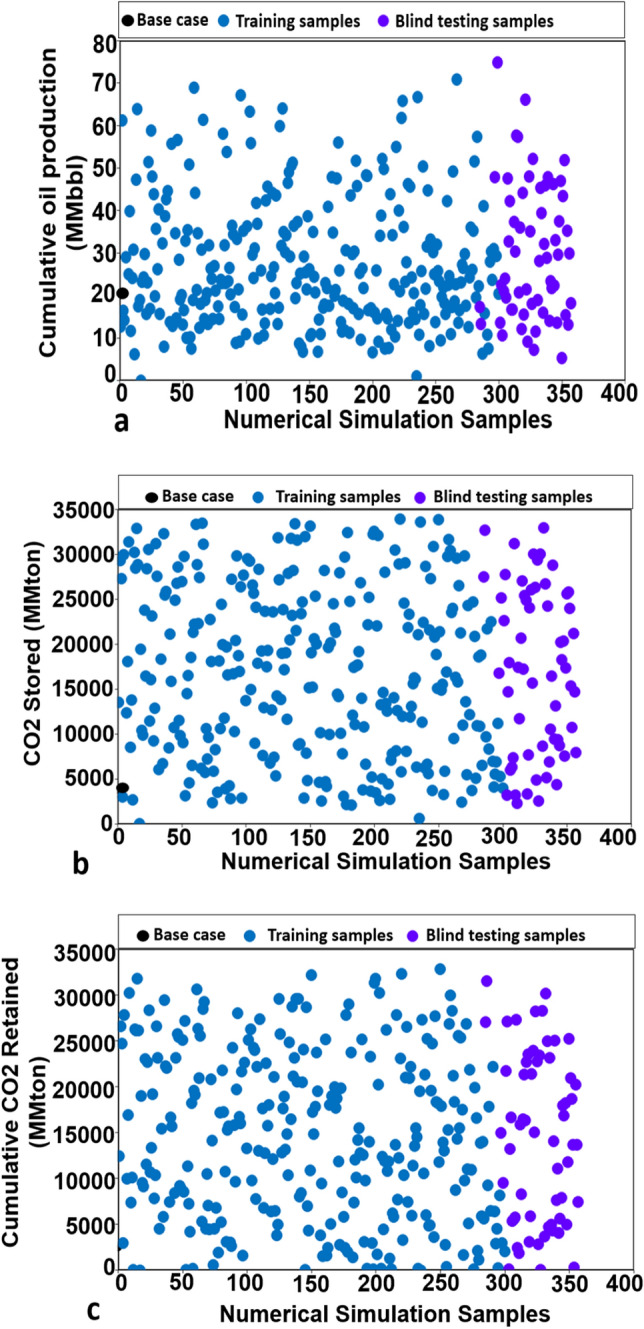
The numerical simulation samples for training and blind testing ANN models. (**a**) The samples of cumulative oil production. (**b**) The samples of cumulative CO_2_ stored. (**c**) The samples of cumulative CO_2_ retained.

Figure 3 highlights the training simulation results of cumulative Oil Production, cumulative CO_2_ retained and the cumulative CO_2_ injection. As depicted in Fig. 3, 351 simulation jobs are diversity in the term of objective functions.

The data-driven model has used 300 samples for a training network. 51 samples were used for a blind testing network to avoid the over-fitting issue. Generally, the ANN model was trained using Levenberg–Marquardt feed-forward back-propagation algorithm. By using MATLAB Network Toolbox, the ANN model was created following an 80%–10%–10% training plan corresponding with the partitioning dataset for a training-validation-test with a total of 300 samples. The same training plan was employed for three different targets. 80% (240 samples) were used for training to calculate the gradient and to update weights and biases. 10% (30 samples) was used for validation to evaluate the network generalization and stop training when generalization halt enhancing. 10% (30 samples) were used for verification to use for comparing different models. The verification scheme is not influencing on “training” therefore; it could evaluate the neural network performance during the training model.

### Optimal number of neurons and hidden layers

Neurons (nodes) are the computational unit that is transfer function to link the input and output connection in ANN. Also, the hidden layer in an ANN architecture is the layer between the input and output layers. The nodes pull a set of weighted uncertainty parameters and output oil production, CO_2_ storage, and CO_2_ retained through activation function in neural networks. Therefore, the number of hidden layers and the number of neurons in each hidden layer are the crucial factors that affect the predictive performance.

**Figure 4:**
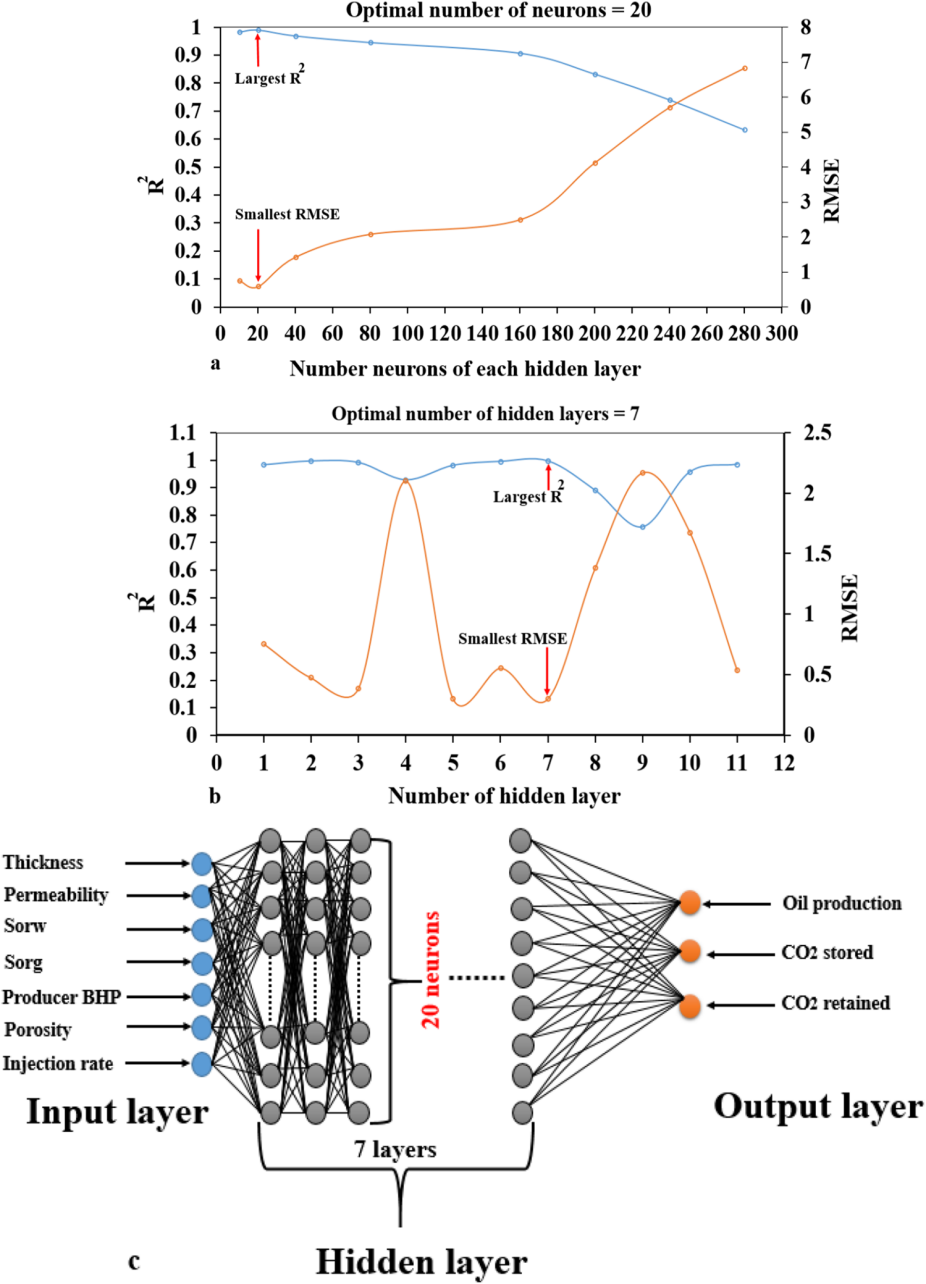
The effect of neuron and hidden layers to ANN models. (**a**) The optimal neurons of each layers. (**b**) The optimal number hidden layer. (**c**) The optimal architecture of ANN prediction models.

Generally, there are several studies to apply ANN for predicting the performance of CO_2_-EOR and storage^34,36^. These studies did not propose the optimal number of neurons and hidden layers. In their works, the number of neurons in single hidden layers is 10 neurons. There is no reason to claim that 10 neurons and one hidden layer are the best solutions for ANN models. Thus, this study addressed this issue to clarify the importance of the number of neurons and hidden layers. The developed ANN model has changed the size of neurons (i.e., 10, 20, 40, 80, 160, 200, 240, 280, 320, 360, 400, 440, 480, 520, 540). Also, the number of hidden layers has changed (i.e., 1, 2, 3, 4, 5, 6, 7, 8, 9, 10, 11). We changed the neurons and hidden layers in ANN models until we obtained the best performance. The result of trial and error to determine the optimal number of neurons and hidden layers highlights in Fig. 4a,b. Besides, Fig. 4c represents the optimal ANN architecture for this study. The performance of this ANN architecture will elaborate in the next section.

### Performance of the ANN model

**Figure 5:**
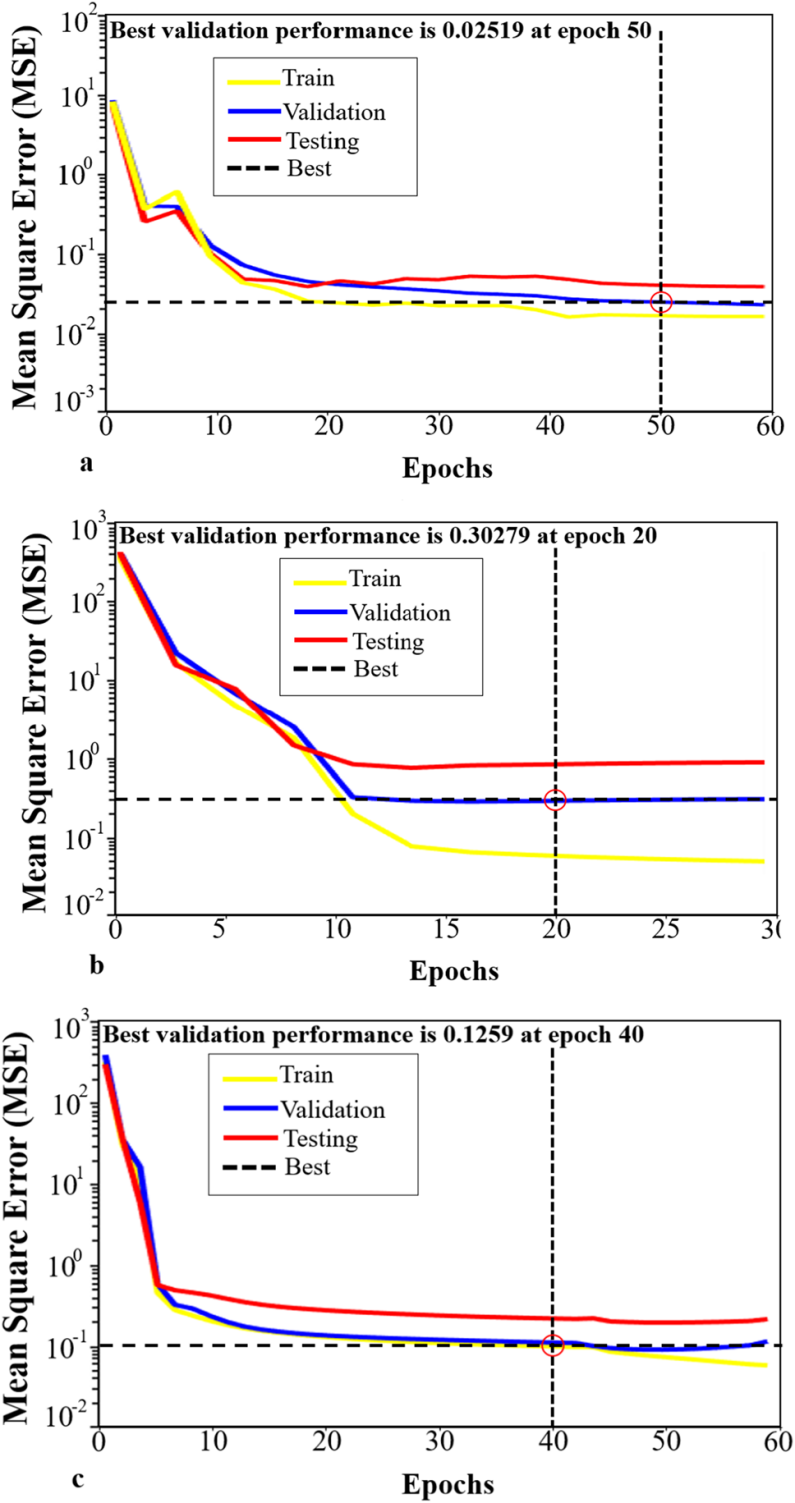
The ANN training performances for three objectives. (**a**) The best validation performance of cumulative oil production is 0.025 at epoch 50th. (**b**) The best validation performance of cumulative CO_2_ stored is 0.3 at epoch 20th. (**c**) The best validation performance of cumulative CO_2_ retained is 0.12 at epoch 40th.

The result of the training performance of three objectives with the mean square error and the number of epoch during the training network depicts in Fig. 5. Successful training is completed until the lowest errors in the verification, and testing curves are nearly similar based on the epoch numbers. As shown in Fig. 5a, the result of cumulative oil production converged to a mean square error of 0.02519 at the 50th iteration. For the cumulative CO_2_ injection and cumulative CO_2_ retained (Fig. 5b,c), the best validation performance is 0.30279, 0.1259 at the 20th and 40th iteration, respectively.

**Figure 6:**
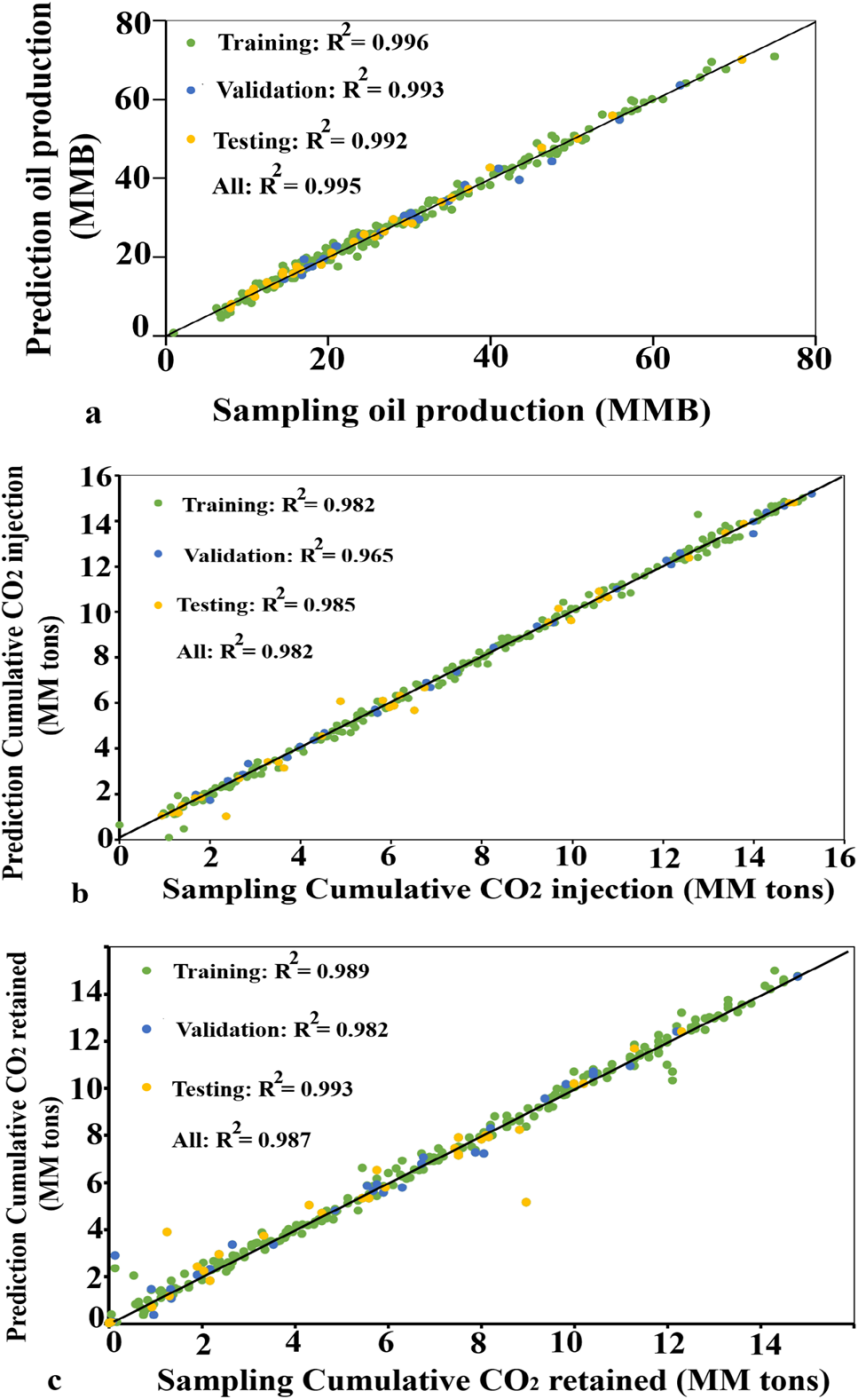
The regression plots comparing the numerical samples and ANN prediction. (**a**) The excellent correlation of training, validation, and testing of cumulative oil production with R^2^ greater than 0.99. (**b**) The R^2^ greater than 0.99 indicated the good performance of cumulative CO_2_ stored network. (**c**) The greater 98% R^2^ for all training, validation, and testing demonstrates the cumulative CO_2_ retained networks.

It is indicated that the training results are reasonable to qualify the following criteria: (i) the mean square error value is small; (ii) the testing curve and verification curve are not very different; (iii) no significant overfitting has occurred during the training process. Moreover, Fig. 6 depicts the excellent correlation between numerical samples and ANN prediction objectives that represented for training-validation-testing data.

As can be seen in this figure, data-driven ANN models for cumulative oil production (Fig. 6a), cumulative CO_2_ stored (Fig. 6b), and cumulative CO_2_ retained (Fig. 6c) were revealed that the overall R^2^ greater than 0.98. This correlation factor proves a similarity between the result of the data-driven ANN model and the numerical simulation value. The R^2^ values and RMSE for the data-driven ANN model are listed out detail in Table 2.

**Table 2:** The correlation factor and Root Mean Square Error for data-driven ANN models.

Parameters	Oil production	CO_2_ stored	CO_2_ retained
R^2^ (overall )	0.995	0.982	0.987
RMSE	1.29	1.44	5.51

Although the ANN prediction model has excellent performance in the term of RMSE and R^2^. It is necessary to test the developed ANN models with blind datasets before employing the predictive model for real fields in the Permian Basin. 51 numerical simulation samples were used to test the data-driven model. These samples were not used during the training process. The blind set data was a matrix of 41 rows and 8 columns.

**Figure 7:**
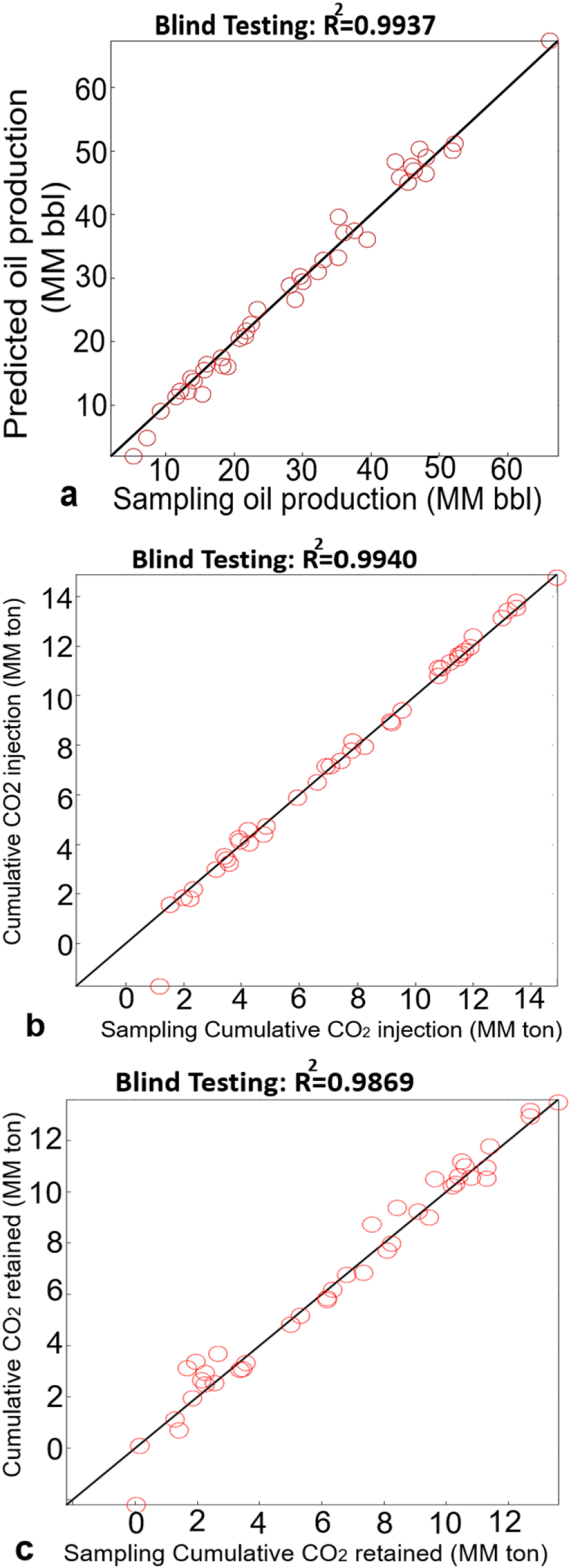
The blind testing of the data-driven ANN models. (**a**) The blind testing result of cumulative oil production with R^2^ greater than 0.993. (**b**) The excellent blind testing of cumulative CO_2_ stored with R^2^ greater than 0.994. (**c**) The greater 98% R^2^ for the blind testing of cumulative CO_2_ retained.

Figure 7 depicts the result of the sample blind test results for cumulative oil production, the cumulative CO_2_ stored, and cumulative CO_2_ retained. The R^2^ greater than 0.98 illustrates the success of blind testing validation for three data-driven models based on ANN in ROZs. These data-driven models will be used for comparative study in the real fields of the Permian basin (USA).

### Field application of ANN model

We deployed the data-driven model created using ANN to several ROZ fields in the Permian Basin. The five fields comprised Robertson (San Andres), Vacuum (Grayburg/San Andres), Wasson (Bennett Ranch), Wasson (Denver), Seminole (San Andres). Table 3 summarizes the values of the reservoir parameters for these fields.

**Table 3:** The residual oil zones parameters for five fields in the Permian Basin, USA.

Parameters	Robertson (San Andres)	Vacuum (Grayburg/San Andres)	Wasson (Benett Ranch)	Wasson (Denver)	Seminole (San Andres)
Thickness (ft)	65	194	150	150	100
Permeability (mD)	15	15	11	12	15
S_org_	0.35	0.314	0.35	0.35	0.32
S_orw_	0.15	0.15	0.15	0.15	0.15
Area (acres)	6000	19,200	7027	27,848	15,700

However, the residual oil saturation to gas (S_org_) for the five fields was not mentioned in the previous study. Thus, we suppose that the S_org_ for all five fields is similar to the Goldsmith field in San Andres area. Therefore, the S_org_ defined as equal to 0.15. Also, Chen and Pawar^7^ confirmed that the cumulative oil production, cumulative CO_2_ injection, and cumulative CO_2_ retained are not so sensitive to S_org_. Therefore the assumption value of S_org_ will not affect the prediction results of data-driven models. Note that the predictive data-driven models are mainly based on the base case model illustrated in Fig. 1b, and the area of the base case reservoir model is 435 acres.

**Figure 8:**
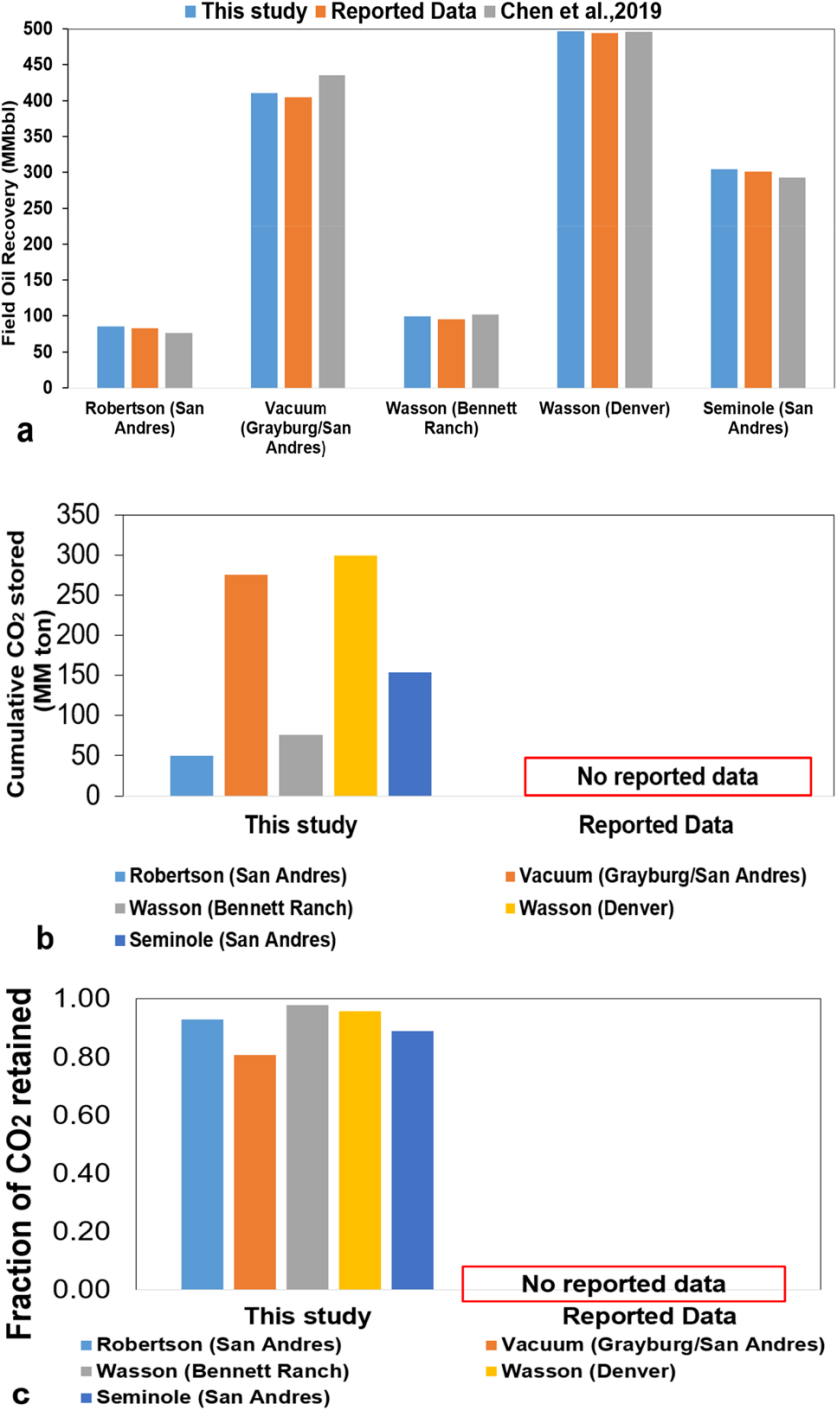
The prediction results of data-driven ANN models for field application in the Permian Basin. (**a**) Comparing between oil production calculated from ANN model and the values recorded in the study of Koperna and Kuuskraa (2006) and Chen et al. (2019). (**b**) The predicted cumulative CO_2_ stored from ANN models. (**c**) The predicted fraction of CO_2_ retained from ANN models.

For each ROZ field with a specific area, the prediction has used the values calculated from the base case reservoir model multiplied by the ratio of the real field area to the base case reservoir model area. For instance, the area of the Robertson field in the San Andres Unit is 6000 acres. The ratio of the real field area for Robertson (San Andres) to the base case reservoir model area is 6000/435 = 13.79. Therefore, the total capacities for Robertson (San Andres) are calculated by multiplying the results predicted by the base data-driven model by the area ratio 13.79. Figure 8a highlights the results of oil recovery for all five ROZ fields computed from the data-driven models and the equivalent values reported in the study of Koperna and Kuuskraa^22^. Figure 8a also depicts the results of oil field recovery for five fields using the ANN model, the predicted results of Chen et al.^7^ and the findings recorded by Koperna and Kuuskraa^22^. Recap that the bottom hole pressure for production wells is set equal to 800 psi and the amount of CO_2_ injection is set equivalent to one million tons per year. We can observe that the prediction results of oil recovery by ANN model closer to the report data than Chen’s study. These results suggested that the ANN models could predict the oil recovery performance with high accuracy in ROZs. By comparison plot, the prediction results ANN models with report data and previous study, we demonstrated that the developed ANN model was enhanced than previous machine learning model.

Moreover, the developed ANN models have also used for evaluating the CO_2_ storage capacity, and the fraction of CO_2_ retained all fives field ROZs. The prediction results are depicted in Fig. 8b,c. As can be seen in Fig. 8b, the highest CO_2_ storage capacity is in Wasson (Denver Unit), and the smallest CO_2_ storage capacity is in Robertson (San Andres Unit). In sum, the potential CO_2_ storage is in two fields: Wasson (Denver Unit) and Vacuum (Grayburg/San Andres). The reason for that is due to the ROZ thickness and area of two fields larger than the remaining fields. The last predictive objective of this work is the fraction of CO_2_ retained in ROZs. We can see from Fig. 8c that the highest fraction of CO_2_ retained in Wasson (Bennett Ranch). Also, reservoir simulation samples were performed using continuous CO_2_ injection with the five-spot well pattern. It was proven that this scenario had the highest amount of CO_2_ storage because the CO_2_ injection did not break through the production well within 10 years oil production period that led to a very high fraction of CO_2_ retained for all five ROZs fields. The prediction results demonstrate that the proposed ANN models can apply for feasibility studies on CO_2_-EOR and storage performance in the field scale CCUS project as well as the Permian Basin. It is indispensable to conduct the preliminary assessment of the potential geological storage formation in the early stage of the CCUS project. The ANN models can predict the level field oil recovery, CO_2_ stored and CO_2_ retained in ROZs with high accuracy by using seven uncertainty parameters, such as thickness, porosity, and permeability, residual oil saturation to water flooding, residual oil saturation to gas flood, CO_2_ injection rate, and producer bottom well pressure. Thus, developed ANN models could consider as a useful, fast, and robust tool to estimate the feasibility of Carbon Capture, Utilization, and Storage (CCUS) projects, especially in ROZs.

### Application perspective of developed ANN models

Although the field application was demonstrated the effectiveness of developed ANN models, however, the unstable oil price is a substantial barrier for CO_2_-EOR and storage project. Therefore, the applicable ANN models should be considered in both technical and economic aspects. In this section, the developed ANN models will serve as the replication-competent of the reservoir simulation model to support for the optimization process.

The CO_2_ injection process will be optimized using Particle Swarm Optimization (PSO) and developed ANN models to obtain the best oil production, CO_2_ storage, and economic parameters such as the Net-Present Value (NPV). The financial metrics for CO_2_ injection were highlighted in Table 4^52^. Besides these economic parameters, the predicted oil prices from 30 to 60 $/bbl was considered for uncertainty in the crude oil market of any CO_2_-EOR project.

**Table 4:** The fiscal and economic parameter for CO_2_ injection project.

Parameters	Values
Oil price	30–60 ($/bbl)
CO_2_ capture	2.38 ($/Mcf)
CO_2_ separation	0.35 ($/Mcf)
Compression	0.65 ($/Mcf)
Transportation	1.16 ($/Mcf)
Injection	0.26 ($/Mcf)
Discount rate	15%
Income tax	30%

**Figure 9:**
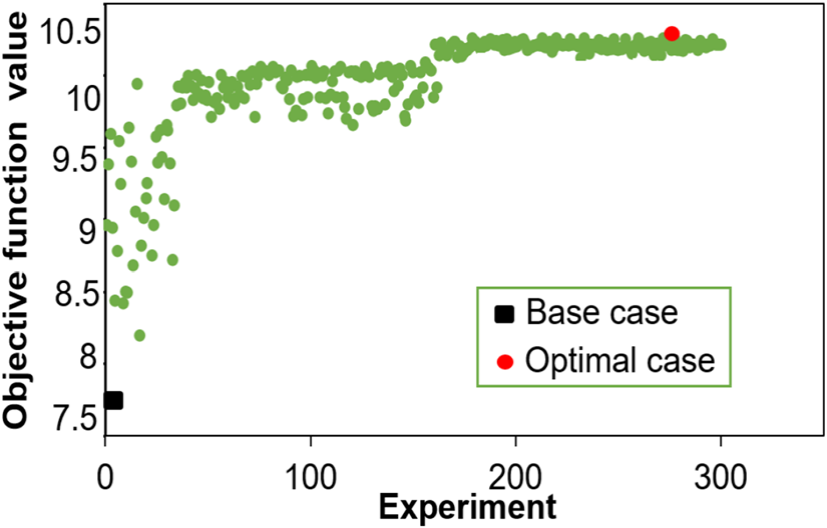
Value of objective function for optimization study.

The PSO approach is used 300 experiment jobs to search for the optimal solution over all of the objective functions. Figure 9 depicts the results of 300 experiment jobs. This figure has also highlighted the performance of the base case and the optimal solution for this work. As can be seen in Fig. 9, the optimal solution is achieved at experiment 278th with the highest value function value of 10.46. Recap that when experiment jobs are over 220th, the objective function values converge to a plane. It is indicated that these 300 experiments already find enough solution distance to achieve the optimal solution.

**Figure 10:**
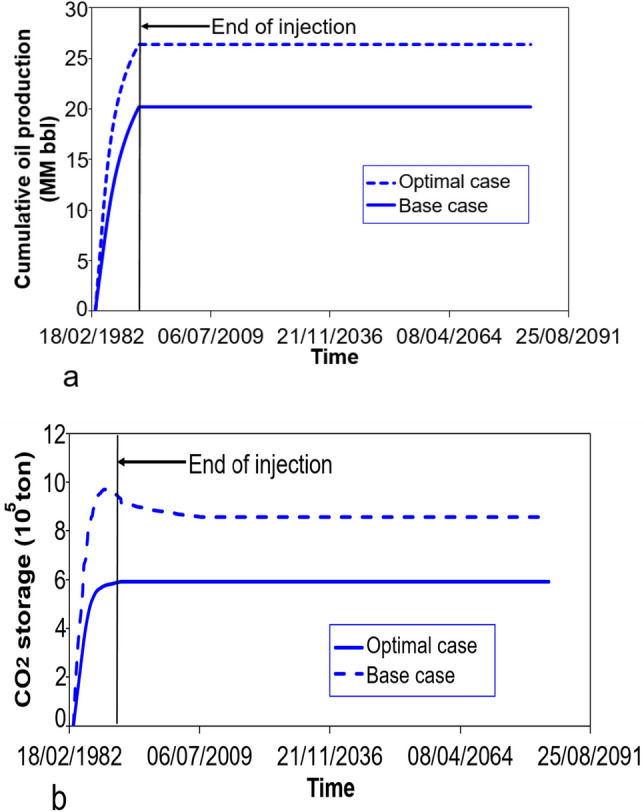
The cumulative oil production for the base case and optimal case (**a**); the CO_2_ storage amount for the base case and optimal case (**b**).

The optimal results of cumulative oil production and CO_2_ storage is highlighted in Fig. 10. It is found that the optimal case has 857,000 tons of CO_2_ storage. The cumulative oil production has 26.4 MM bbl.

For better evaluation of the improvement of the optimization process, the base case scenario was compared with the optimal solution. The optimization results shown that the cumulative oil production was increased by 30.60% and NPV of optimal case was enhanced by 33.08%. Also, the CO_2_ storage had been improved by 44.76%. At the same time, the objective function was enhanced from 7.63 to 10.46. Table 5 summarizes the comparison of the baseline case and optimal case.

**Table 5:** Comparison between the base case and the optimal solution.

Results	Unit	Base case	Optimal case	Difference (%)
Cumulative oil production	10^7^ bbl	2.02	2.64	30.60
CO_2_ storage	10^5^ ton	5.92	8.57	44.76
NPV	10^7^ USD$	2.66	3.54	33.08
Objective function	10^7^ bbl oil + 10^7^ lbmol CO_2_ + 10^7^$	7.63	10.46	37.09

**Figure 11:**
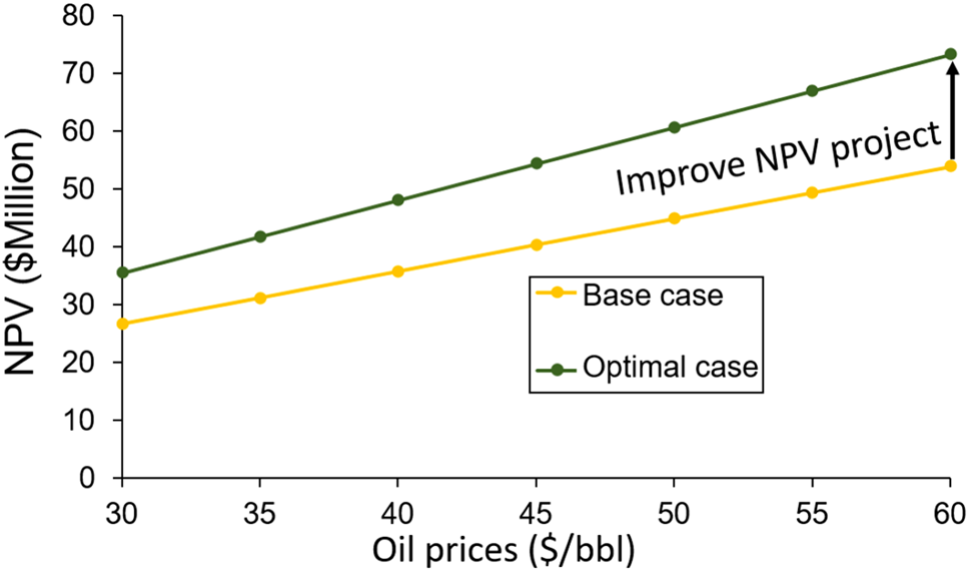
NPV comparison between the base case and optimal solutions achieve with different values of oil prices.

The optimal solution in this study had been better prediction performance than the base case scenario in all considered objectives, including cumulative oil production, CO_2_ storage, and Net Present Value. Furthermore, the role of developed models was integrated with PSO to speed up the optimization process. The PSO coupling ANN models need only 566 s to obtain the optimal solution that reducing computational time for the optimization process. Also, this study was considered the unstable oil prices to evaluate the NPV projects. Utilization of the ANN models, the base case and optimal case were economically calculated the NPV with the range of oil prices vary from $30–$60/barrel, as highlighted in Fig. 11.

It can be seen in Fig. 11 that the optimal solution demonstrates more feasible economic consideration for a range $(30–60) per barrel oil prices. This result indicates the excellent perspective of developed ANN models for evaluating the economic feasibility of a project. Furthermore, these developed ANN models would provide a fast and robust tool to estimate project economic benefits.

## Discussion

Our results suggest that the need to develop the ANN predictive tools for evaluation of the performance of CO_2_-EOR and storage in ROZs. We showed that the ANN models could achieve the high accuracy of prediction results by comparing it with reported data from five real fields in the Permian Basin. The reason for this excellent predictive performance because of the careful selection of the specific uncertainties parameters for training ANN models. Also, the blind testing process is fundamental to verify the accuracy of ANN models. Many studies using ANN without consideration of the blind testing network. This issue should not ignore when we developed the ANN models.

We recognized that each ANN model is case-specific, which means the ANN model built for one particular area cannot be used in another reservoir characteristic. For instance, this study was developed for ROZs, so the ANN model from this work just applied in the ROZs field. However, the procedure is generated the DDM is easy to adapt for case by case. Also, the key element to producing the ANN model is the spatiotemporal database training. The more reservoir information comprised in the training database, the more accuracy the network training will be. We can adjust the number of reservoir parameters for “training” ANN model depended on the available information. Furthermore, the range of uncertainty parameters used in training models is significant. The selection specific range for training cases should refer to the literature or previous work. The ANN models might not give robust results if the models are tested on the properties out of the range of training cases.

Our findings find that the number of simulation jobs is an important component for hand-shaking reservoir simulation and machine learning tools to develop the ANN models.

This work was used 351 samples for training and blind testing network purpose. Note that increasing the numerical samples led to improve predictive models. Thus, the difference between predictive results and field report data is less than 8%. While the other study used 250 samples for a generation, the predictive empirical models to get a difference less than 10%^7^. The issue of the numerical samples was not raised when using ANN for predictive the performance of CO_2_-EOR and storage^22,36^. Also, these past studies did not pay attention to verification sample blind test results. However, our work was clearly expressed the performance of predictive models before “employ” the ANN model for prediction in the real field application.

Furthermore, our study was demonstrated the application perspective of the developed ANN model by coupling with PSO to speed up the optimization process. The advantage of ANN models could support conventional reservoir simulator to reduce time-consuming for engineering applications such as sensitivity analysis and optimization aspect.

In summary, our study proposes an innovative framework for “generation” robust and high accuracy ANN models. In order to reproduce the proposed method, it is recommended hitherto for other CO_2_ storage “formation” such as saline aquifers, depleted hydrocarbon reservoirs, and unconventional reservoirs. A probable limitation of data-driven models is applicable to different geology characteristics. However, our work claims that the selection uncertainty variables for the training scheme would reduce the weakness of data-driven models. Therefore, this methodology could be applied in the different aspects of CCUS, Enhanced Oil Recovery, reservoir engineering, and other science disciplines.

## Conclusions

This study assessed the performance of CO_2_-EOR and storage in Residual Oil Zones using Artificial Neural Networks. It explored the applicable of data-driven models for prediction field oil recovery, CO_2_ stored, and CO_2_ retained in the real field ROZs in the Permian basin (USA). The following key points could be drawn based on the findings of this work:

1. Numerical reservoir simulation of residual oil zones was conducted to generate the training database utilized as input and output layer in ANN data-driven model design. This study was created 351 numerical simulation jobs for the spatiotemporal database to collect the objective function included CO_2_ oil production, CO_2_ stored, and CO_2_ retained in the ROZs reservoir model.
2. The developed ANN model was built with the optimal design of architecture comprising of 7 hidden layers and 20 neurons of each hidden layers , minimum Mean Square Error, the maximum correlation factor (R^2^) of testing data set. As the verification, the blind testing results revealed that the R^2^ was higher than 0.99, and the overall training had a low MSE of less than 2%.
3. As a real field application, data-driven models were applied for five ROZs fields in the Permian basin, USA. We found that the ANN models can achieve an excellent forecast of oil recovery that fits excellent with the report data conducted by Koperna and Kuuskraa^22^. Furthermore, the ANN models can also be adapted to predict the CO_2_ storage capacity in multiple ROZs. The excellent agreement of the predictive CO_2_ stored results was compared with the data in the work of Chen and Pawar^7^. Also, to the best of our knowledge, the CO_2_-EOR and storage capacity has not been investigated in ROZs by using data-driven ANN models.
4. The proposed ANN models can predict the CO_2_-EOR and storage performance with high accuracy in ROZs. Our findings suggest that the developed models can be reproduced and applied in the other aspect of EOR and CO_2_ sequestration, such as prediction trapping index, CO_2_ leakage from the cap-rock or CO_2_ plume migration area. Besides, the proposed data-driven modelling workflow is supposed very useful in researches and practical applications, especially the intelligence techniques that are commonly developed and utilized.
5. The proposed data-driven model can be linked with commercial simulation packages such as CMG or ECLIPSE to enhance their ability and accuracy for forecasting the CO_2_-EOR and storage performances in geological formations.
6. The developed ANN models could integrate with other optimization algorithms to improve the speed of the optimization process.
